# Machine Learning Model for Predicting Multidrug Resistance in Clinical *Klebsiella pneumoniae* Isolates

**DOI:** 10.3390/diagnostics16040555

**Published:** 2026-02-13

**Authors:** Yuksel Akkaya, Irfan Aydin, Handan Tanyildizi-Kokkulunk, Ayse Erturk, Ibrahim Halil Kilic

**Affiliations:** 1 Department of Medical Microbiology, Hamidiye Faculty of Medicine, University of Health Sciences, Istanbul 34668, Türkiye; 2 Pharmacy Services Program, Pharmacy Services Department, Health Services Vocational School, Fenerbahce University, Istanbul 34758, Türkiye; irfan.aydin@fbu.edu.tr; 3 Department of Arts & Sciences, Maine Maritime Academy, Castine, ME 04420, USA; handan.kokkulunk@mma.edu; 4 Department of Microbiology and Clinical Microbiology, Dr. Siyami Ersek Thoracic and Cardiovascular Surgery Education and Research Hospital, Istanbul 34668, Türkiye; ayse.erturk3@saglik.gov.tr; 5 Department of Biology, Faculty of Arts and Sciences, Gaziantep University, Gaziantep 27410, Türkiye; kilic@gantep.edu.tr

**Keywords:** antibiotic resistance, clinical decision support system, *Klebsiella pneumoniae*, machine learning, random forest

## Abstract

**Background/Objectives:** *Klebsiella pneumoniae* is an opportunistic pathogen increasingly resistant to carbapenems and broad-spectrum antibiotics, complicating timely infection management. In critical cases like septic shock, where initiating effective antibiotics within 3 h improves survival, culture-based resistance testing is often too slow. This study evaluates machine learning (ML) algorithms for faster antimicrobial resistance prediction than conventional methods. **Methods:** In this retrospective study, antibiogram results of 607 *Klebsiella pneumoniae* isolates collected between 2017 and 2024 were combined with demographic and clinical information of the patients from whom the isolates were obtained. Four different ML algorithms, namely Decision Tree (DT), Support Vector Classifier (SVC), K-Nearest Neighbors (KNN) and Random Forest (RF), were applied to classify the resistance status for 22 antibiotics. Model performances were evaluated using accuracy, precision, recall, F-score, AUC and feature importance metrics. **Results:** The RF model showed the highest overall performance in accurately predicting resistance to 22 antibiotics, achieving an average AUC value of 0.96. In particular, it predicted resistance to treatment-critical antibiotics such as Ertapenem (100%), Imipenem (93%) and Meropenem (95%) with high accuracy. **Conclusions:** ML models, especially RF, offer a powerful tool for rapid antibiotic resistance prediction, supporting accurate empirical treatment decisions and antimicrobial stewardship.

## 1. Introduction

*Klebsiella pneumoniae* is the most common member of the Klebsiella genus, belonging to the Enterobacteriaceae family, and can survive under aerobic or facultative anaerobic conditions as a Gram-negative bacterium [1]. This opportunistic pathogen is widely distributed in the environment and can also colonize healthy individuals, and it is responsible for a broad range of infections, including respiratory and urinary tract infections, meningitis, joint and gastrointestinal infections, as well as septic complications. Among the 12 identified Klebsiella species, *Klebsiella pneumoniae* accounts for approximately 85% of clinical isolates [2].

According to the Global Burden of Disease study published in 2022, antimicrobial resistance (AMR) was identified as a leading cause of death globally, contributing to approximately 4.95 million deaths associated with resistant infections, of which 1.27 million were directly attributable to AMR [3]. *Klebsiella pneumoniae*, along with *Escherichia coli*, *Staphylococcus aureus*, *Streptococcus pneumoniae*, *Acinetobacter baumannii*, and *Pseudomonas aeruginosa*, was highlighted as one of the primary pathogens responsible for 73% of these AMR-related fatalities [3]. Furthermore, the World Health Organization (WHO) has categorized carbapenem-resistant Enterobacteriaceae, including *Klebsiella pneumoniae*, within the ‘critical priority’ tier of its Global Priority Pathogens List (PPL) to guide the research and development of new antibiotics [4]. The high prevalence of multidrug resistance among ESKAPE pathogens, including *Klebsiella pneumoniae*, remains a dominant determinant of mortality, particularly when inappropriate empirical therapy is initiated [5].

The impact of AMR is particularly severe in immunocompromised populations, children under 5 years of age, and the elderly, where it poses significant challenges for treatment planning. Antimicrobial resistance also leads to a significant increase in health expenditures due to prolonged intensive care stays, extensive diagnostics, costly treatments, disability, and mortality costs [6]. In 2015, the World Health Organization (WHO) launched the Global Antimicrobial Resistance and Use Surveillance System (GLASS), the first global collaborative initiative to standardize AMR surveillance through data collection, analysis, interpretation, and sharing [7]. This system aims to promote evidence-based prescribing, optimize clinical outcomes, and reduce healthcare costs. Therefore, AMR surveillance data can effectively impact mortality and costs by guiding empirical antimicrobial therapies and reserving last-resort antibiotics for specific infections [6].

Its rising antibiotic resistance has become a major global concern, with reported rates of 68% in South Africa [8], 54% in India [1], 97% in Equatorial Guinea [9], and 53% in Ethiopia [10]. Conventional antibiogram tests require several days, delaying appropriate therapy and leading to higher costs and mortality. Machine learning (ML) offers promising applications in healthcare, including disease diagnosis and treatment prediction. By analyzing antibiotic susceptibility profiles and clinical data, ML models can uncover patterns often missed by traditional statistical approaches [11,12,13,14,15].

In this study, we evaluated the performance of four widely utilized supervised learning architectures, Decision Tree (DT), K-Nearest Neighbors (KNN), Support Vector Classifier (SVC), and Random Forest (RF), to analyze high-dimensional clinical data and optimize antimicrobial resistance detection strategies.

Summary of Contributions: This study extends beyond phenotype-specific limitations (e.g., Carbapenem resistance) by introducing distinct predictive models for 22 antibiotics, thereby broadening the scope for empirical therapy guidance. By calibrating decision thresholds via Youden’s J statistic, the methodology prioritizes sensitivity and Negative Predictive Value to reduce false negatives, addressing the safety requirements of clinical practice. Furthermore, the model simulates a “Partial Antibiogram” workflow, using cross-resistance patterns to predict pending results from early data without leakage. Finally, feature analysis identifies temporal trends and co-resistance clusters as the primary determinants of prediction, shedding light on local resistance dynamics.

## 2. Materials and Methods

This study was approved by the Scientific Research Ethics Committee of the University of Health Sciences Ümraniye Training and Research Hospital (approval no. B.10.1.TKH.4.34.H.GP.0.01/202, 19 June 2025). All procedures complied with institutional and national ethical guidelines, as well as the 1964 Helsinki Declaration and its later amendments. The study applied ML algorithms to antibiogram results and clinical data of *Klebsiella pneumoniae* strains isolated from samples processed in the microbiology laboratory of Dr. Siyami Ersek Thoracic and Cardiovascular Surgery Training and Research Hospital between 1 January 2017 and 1 January 2024. Strain identification was performed using MALDI-TOF MS (VITEK MS v.3.2, bioMérieux, Marcy-l’Étoile, France). Antimicrobial susceptibility testing (AST) was conducted using the VITEK^®^ 2 Compact system (software version 9.02, bioMérieux, Marcy-l’Étoile, France). This system utilizes an automated kinetic measurement principle, monitoring bacterial growth through turbidimetric and colorimetric analysis every 15 min to determine minimum inhibitory concentrations (MICs).

### 2.1. Dataset Information

Information on the grouping of the data we used in our study between 1 January 2017 and 1 January 2024 and the number of samples is given in Table 1.

### 2.2. Data Cleaning and Preprocessing

To ensure statistical reliability and minimize noise from sparse data, patients with fewer than 10 tested antibiotic records were excluded. This filtering criterion was adopted to avoid unstable or biased estimates associated with insufficient class diversity, reducing the dataset from 761 to 607 patients. No missing values were observed in demographic or clinical variables (gender, year, department, sample type). However, among the 22 antibiotics (Table 2), 2661 of 23,673 data points (representing sporadic untested antibiotics in the panel) were missing (≈11%). These were imputed using a patient-based mode substitution, where the most frequent value for each patient’s antibiotic profile was applied.

For the remaining ≈11% missing antibiotic susceptibility data, a patient-mode imputation strategy was used, assigning each patient the most frequent resistance status observed within their available antibiotic profile. This approach was selected to prevent the reduction in statistical power associated with listwise deletion and is grounded in the biological nature of *Klebsiella pneumoniae*, where resistance phenotypes are highly correlated due to shared mechanisms (e.g., extended-spectrum *β*-lactamase or carbapenemase activity) and plasmid-mediated co-transmission of genes (e.g., MDR clones). Utilizing the most frequent within-patient status leverages this intrinsic biological dependency rather than treating missing values as random noise. Regarding its impact, this method preserved the sample size (*n* = 607) and maintained the structural integrity of the antibiograms, minimizing the selection bias that would arise from excluding patients with incomplete but clinically relevant resistance profiles [1]. Sensitivity analyses comparing patient-mode, multiple imputation, and listwise deletion methods revealed minimal differences (<1% variation in AUC), confirming that model performance was not materially influenced by the imputation method. Given this negligible impact and the biological coherence of intra-isolate resistance patterns, the patient-mode approach provided a justified and computationally efficient means of maintaining dataset completeness without introducing measurable bias.

To address class imbalance, the SMOTE technique was applied to the training set, generating synthetic minority instances and improving decision boundary learning. Model performance was evaluated using five-fold cross-validation to reduce overfitting risk. Since all features were categorical, One-Hot Encoding was performed to enable numerical analysis. The antibiotics studied and resistance distributions are presented in Table 2.

### 2.3. Model Design and Feature Exclusion

In this study, multidrug resistance (MDR) was defined according to the standardized international terminology proposed by the European Centre for Disease Prevention and Control (ECDC) and the Centers for Disease Control and Prevention (CDC). Specifically, MDR was characterized as acquired non-susceptibility to at least one agent in three or more antimicrobial categories [16]. Our dataset included isolates spanning MDR, extensively drug-resistant (XDR), and pandrug-resistant (PDR) profiles, ensuring a comprehensive evaluation of resistance phenotypes [16,17].

To eliminate circular reasoning and data leakage, we adopted a strict “target-exclusion” strategy for every antibiotic-specific model. Rather than relying on a single global classifier for MDR status, we trained 22 independent models corresponding to each target antibiotic. For instance, when predicting resistance to Meropenem, the susceptibility result for Meropenem itself was strictly omitted from the training data.

The input features were structured to reflect a “Partial Antibiogram” scenario, mirroring the clinical reality where results for distinct antimicrobial panels often arrive with latency (e.g., rapid beta-lactams vs. delayed colistin testing). Consequently, the input vector for any given target combined demographic and clinical metadata, such as age, gender, department, sample type, and year, with the co-resistance profiles of the remaining 21 antibiotics. This structure exploits biological correlations found in plasmid-mediated cross-resistance mechanisms as predictive signals, ensuring the ground truth is never used to predict itself. The variable reporting times of the VITEK^®^ 2 system, ranging from 4 to 18 h depending on the isolate’s growth rate and specific drug–organism interactions, create a distinct clinical reporting gap. Our model is specifically designed to function within this window, utilizing early available AST results (often known by hours 6–8) to predict delayed results (typically finalized by hour 18), thereby providing data-driven guidance for empirical therapy during the most critical stages of infection management.

### 2.4. Machine Learning Workflow and Model Validation

#### 2.4.1. Model Selection and Rationale

The classification framework relies on a comparative analysis of four supervised algorithms: Decision Tree (DT), Support Vector Classifier (SVC), K-Nearest Neighbors (KNN), and Random Forest (RF). We prioritized these architectures due to their distinct structural approaches and reliability in processing high-dimensional clinical data, balancing interpretability with performance.

- DT provides high interpretability by mapping decision rules, which is critical for clinical explanation [18].
- SVC is effective in high-dimensional spaces by defining optimal hyperplanes [19].
- KNN offers a non-parametric approach based on feature similarity [20].
- RF was utilized as an ensemble method to reduce the variance of individual trees and prevent overfitting, a common challenge in medical datasets [21].

While deep learning models exist, these classical algorithms were prioritized to balance predictive performance with computational efficiency and the limited sample size (*n* = 607), where deep learning often struggles with overfitting.

#### 2.4.2. Experimental Design and Data Splitting

The dataset was processed using a Python (version 3.13; Python Software Foundation, Wilmington, DE, USA) based pipeline (libraries: NumPy, Pandas, Scikit-learn). Prior to any analysis, the full dataset (*n* = 607) was partitioned into a training set (80%, *n* = 485) and an isolated hold-out test set (20%, *n* = 122). A fixed random seed (random_state = 42) was used to guarantee reproducibility, ensuring the test set remained completely separate from the training and hyperparameter tuning phases. Following the split, the preprocessing pipeline, comprising One-Hot Encoding for categorical data and patient-mode imputation for missing values, was fitted solely on the training folds. These parameters were then applied to the test data, thereby preventing data leakage and simulating a realistic deployment environment.

#### 2.4.3. Handling Class Imbalance (SMOTE)

Since the dataset contained uneven resistance rates, such as Colistin at approximately 31%, there was a risk of the models favoring the majority class. To mitigate this bias, we implemented the Synthetic Minority Oversampling Technique (SMOTE). We were careful to apply this augmentation strictly to the training data within each cross-validation fold, leaving the test set completely untouched. In practice, this step balanced the class distribution effectively; for instance, the minority count for Colistin in a typical training fold rose from 152 to 334. We retained this step in the final pipeline after preliminary tests confirmed that oversampling led to a marked improvement in sensitivity (Recall) for the minority resistance phenotypes. Specifically, the model trained without SMOTE exhibited a critically low sensitivity of 38% for the minority Colistin class; however, the integration of SMOTE increased this to 73% without compromising specificity, thereby fulfilling the clinical safety requirement.

#### 2.4.4. Hyperparameter Tuning (GridSearchCV)

To maximize model performance and avoid relying on default settings, hyperparameters for all four algorithms were optimized using GridSearchCV with 5-fold cross-validation. The search space included:

- RF: n_estimators (100, 200, 500), max_depth (None, 10, 20), min_samples_split (2, 5).
- SVC: C (0.1, 1, 10), kernel (‘linear’, ‘rbf’).
- KNN: n_neighbors (3, 5, 7, 9), weights (‘uniform’, ‘distance’).
- DT: criterion (‘gini’, ‘entropy’), max_depth (None, 10, 20).

The optimal parameters identified by GridSearchCV were used to train the final models on the full training set.

#### 2.4.5. Feature Selection and Prevention of Data Leakage

The study utilized 22 individual models instead of a broad MDR classifier. To ensure validity, we enforced a protocol where the target antibiotic’s result was excluded from its own prediction features. The input space was constructed using demographic variables (Age, Gender, Department, Sample Type, and Year) alongside the susceptibility status of the other 21 agents in the panel. This design leverages cross-resistance patterns to mimic a “Partial Antibiogram” workflow, predicting missing values without any data leakage.

#### 2.4.6. Performance Evaluation

The independent test set served as the benchmark for evaluating Accuracy, Precision, Recall, F-score, and AUC. To derive binary classifications, we calibrated the optimal cutoff points using Youden’s J statistic (J = Sensitivity + Specificity − 1) instead of a fixed 0.5 threshold. By focusing on the point where the sum of sensitivity and specificity is maximized (J), this approach optimizes the trade-off between detecting resistance and ruling out susceptible cases [22].

### 2.5. Declaration of Generative AI in Scientific Writing

During the preparation of this study, a generative artificial intelligence tool (Gemini 1.5 Pro Google, Mountain View, CA, USA) was utilized exclusively for the visual conceptualization and design of the Graphical Abstract. The authors emphasize that no GenAI tools were used for study design, data collection, analysis, interpretation, or the generation of the manuscript’s text. The authors have reviewed and edited the graphical output and take full responsibility for the content of this publication.

## 3. Results

Optimal hyperparameters were established via GridSearchCV prior to testing. The final Random Forest configuration comprised 200 estimators with unrestricted depth (max_depth = None), while the SVC achieved optimal separation using an RBF kernel (C = 10). For KNN, performance was maximized at k = 5 with distance weights. Furthermore, the use of Youden’s J statistic replaced standard boundaries with dynamic probability thresholds (Range: 0.38–0.62; Median: 0.48). This adjustment tuned the models to be more sensitive to resistant cases, directly influencing the high detection rates observed in the study.

**Figure 1:**
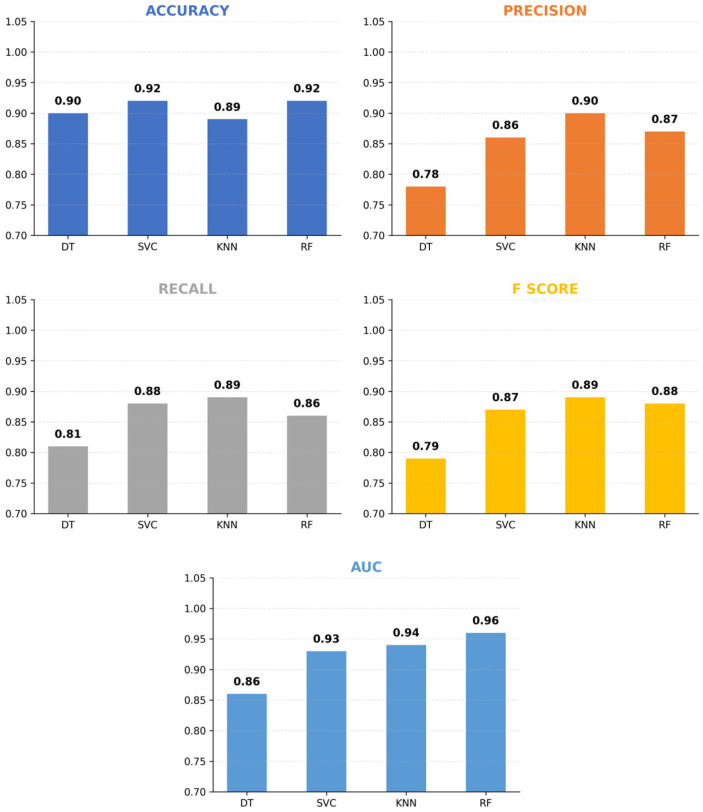
Key performance evaluation metrics calculated for DT, SVC, KNN and RF algorithms.

This study evaluated antibiotic resistance data from 607 patients using ML classifiers to predict resistance to 22 antibiotics. The close alignment of training and test performance indicated no underfitting or overfitting, suggesting a balanced trade-off between bias and variance. Overall, the models demonstrated robust predictive capacity, with Random Forest achieving the highest AUC across antibiotics (Figure 1).

In the performance evaluation of the applied ML algorithms, the DT model yielded an accuracy of 0.90 ± 0.072, an AUC value of 0.86 ± 0.089, a precision of 0.78 ± 0.158, a recall of 0.81 ± 0.161, and an F-score of 0.79 ± 0.158. The SVC achieved an accuracy of 0.92 ± 0.064, an AUC of 0.93 ± 0.076, a precision of 0.86 ± 0.196, a recall of 0.88 ± 0.203, and an F-score of 0.87 ± 0.196. The KNN algorithm achieved an accuracy of 0.89 ± 0.069, an AUC of 0.94 ± 0.048, a precision of 0.90 ± 0.059, a recall of 0.89 ± 0.069, and an F-score of 0.89 ± 0.065. Last, the RF model demonstrated an accuracy of 0.92 ± 0.065, the highest AUC value of 0.96 ± 0.046, a precision of 0.87 ± 0.197, a recall of 0.86 ± 0.201, and a F-score of 0.88 ± 0.200. These results show that all models performed well, with RF excelling in terms of AUC, while RF and SVC achieved the highest overall accuracy.

Considering the class imbalance present in the dataset used in this study, AUC serves as a more reliable performance metric compared to accuracy. A high AUC value indicates the model’s strong ability to distinguish each class from the others [23]. Therefore, the AUC score of 0.96 obtained for the RF algorithm demonstrates its high discriminative power and effective classification performance across all classes. As a result, the RF model was identified as the optimal algorithm for antibiotic resistance classification. Beyond AUC and accuracy, clinical operating characteristics were also examined. Positive and Negative Predictive Values (PPV and NPV) calculated at optimal cut-off points (Youden’s J statistic) exceeded 0.90 for carbapenem antibiotics, indicating low false-negative rates and high reliability in distinguishing resistant from susceptible isolates. By applying Youden’s J optimization, the models achieved balanced performance metrics, with sensitivities ranging from 73% to 100% across the tested antibiotics, confirming the robustness of the threshold selection. These clinically oriented metrics further confirm the model’s utility for guiding empirical antibiotic selection. The metrics calculated according to the best performing RF model for each antibiotic resistance classification are shown in detail in Table 3.

**Figure 2:**
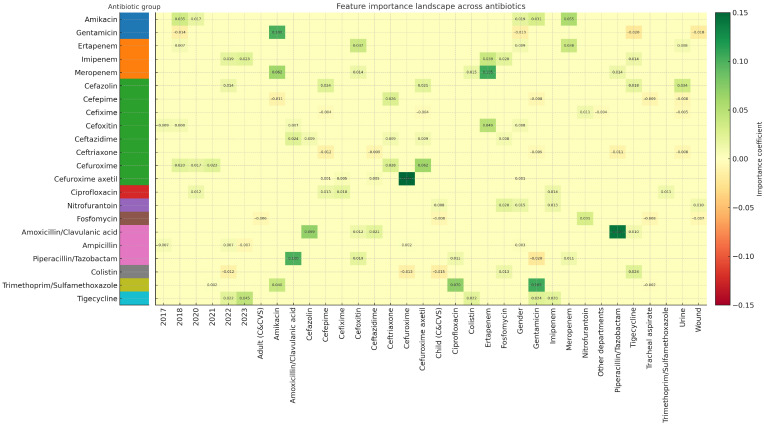
Feature importance landscape across antibiotic-specific resistance models. Note: Heat map presenting permutation-based feature importance values for the Random Forest models predicting resistance against 22 antibiotics. Each cell represents the contribution of a specific clinical or microbiological variable to the prediction performance of a given antibiotic model. Green shades indicate positive influence (higher importance), whereas orange-red shades indicate negative influence. The clustering and color gradient highlight distinct predictor–antibiotic relationships, including multidrug-resistance-associated cross-predictivity and ward- or sample-specific effects. Permutation importance analysis revealed heterogeneous but clinically meaningful predictors of antimicrobial resistance across antibiotics. Temporal variables (particularly year of isolation) demonstrated a strong influence on resistance prediction for multiple antibiotics, including Amikacin, Gentamicin, Ertapenem, Imipenem, Cefazolin, Cefoxitin, Cefuroxime, Ciprofloxacin, Ampicillin, Colistin, Trimethoprim/Sulfamethoxazole, and Tigecycline, suggesting an increasing trend in resistance over time. Patient gender contributed notably to resistance prediction for aminoglycosides, β-lactams, and nitrofurans, while clinical specimen type exhibited selective predictive value: urine for cephalosporins and carbapenems, wound for fosfomycin and gentamicin, and tracheal aspirate for cefepime and trimethoprim/sulfamethoxazole.

The feature importance landscape for antibiotic resistance prediction is illustrated in Figure 2, where the top-ranked predictors for each antibiotic are visualized. A detailed numerical representation of these predictors is provided in the Supplementary File “Feature Importance Table”.

Clinical care settings were also informative; pediatric and adult cardiology/cardiovascular surgery wards emerged among the most influential predictors for colistin, fosfomycin, and nitrofurantoin resistance, reflecting ward-specific antimicrobial exposure patterns. Importantly, resistance to other antibiotics remained the strongest predictor across most models, highlighting the interconnected nature of multidrug resistance and the presence of co-resistance clusters in *Klebsiella pneumoniae*.

Negative importance values were observed for certain predictors, particularly for Gentamicin, Cefepime, Cefixime, Ceftriaxone, Fosfomycin, Ampicillin, Piperacillin/Tazobactam, Colistin, and Trimethoprim/Sulfamethoxazole, indicating that model performance improved when these variables were permuted. This finding underscores the necessity of antibiotic-specific modeling strategies and cautious interpretation of variable effects when developing clinical decision-support tools. Subgroup analysis across various clinical specimens, including wound (28.3%), urine (24.2%), tracheal aspirates (22.2%), and blood (11.7%), revealed that the Random Forest model maintained stable predictive performance across different infection sites. This robustness suggests that the model is versatile enough to be deployed across diverse clinical scenarios without sample-specific bias. The numerical importance rankings for each antibiotic are provided in the Supplementary File “Feature Importance Table”.

## 4. Discussion

The World Health Organization has identified *Klebsiella pneumoniae* as a serious threat to global public health due to its increasing resistance to multiple critically important antimicrobials [24]. This widespread resistance complicates the management of healthcare-associated infections, contributes to higher morbidity and mortality rates, and leads to longer hospitalization times and increased healthcare expenditures [25,26,27]. Therefore, new strategies to efficiently detect and manage resistant *Klebsiella pneumoniae* infections are urgently needed.

Traditional susceptibility testing methods require up to 72 h from specimen collection to final reporting, exposing patients to extended empirical therapy and potentially inappropriate antibiotics during critical time windows [28]. In infections such as septic shock, timely initiation of effective therapy is crucial; each hour of delay significantly decreases survival rates [29]. More rapid identification of resistance patterns would therefore directly improve patient outcomes, support rational antibiotic use, and help reduce the spread of antimicrobial resistance.

ML offers promising solutions by rapidly processing large and complex datasets to provide actionable treatment guidance [11]. Integrating high-performance ML models into clinical microbiology workflows can optimize empirical antibiotic selection, reduce diagnostic turnaround times, and decrease testing and treatment costs [11,30]. Increased digitization of patient information and laboratory systems has improved the feasibility and accuracy of ML-based predictions [30,31]. Notably, even though our approach requires partial AST data as input, it addresses a key clinical gap: some antibiotics (e.g., β-lactams) are typically reported earlier, while others (e.g., colistin) may require additional testing outside automated panels. By inferring pending antibiotic results from early available data, ML can accelerate decision-making when rapid intervention is essential. While this study utilizes data from the VITEK^®^ 2 system, the underlying methodology is platform-agnostic and can be integrated into any automated microbiology workflow that generates asynchronous susceptibility data. This approach demonstrates how routine laboratory data can be transformed into actionable clinical intelligence, regardless of the specific hardware utilized.

The clinical necessity of this acceleration is underscored by the latest ESCMID guidelines, which highlight that for severe infections and bloodstream infections (BSIs) caused by third-generation cephalosporin-resistant Enterobacteriaceae, the timely selection of appropriate agents is critical for survival [32]. Especially in emergency departments, where initial antibiotic choice is arguably the most vital decision for life-threatening infections, our model’s ability to bridge the reporting gap aligns with ‘Antimicrobial Stewardship’ goals of optimizing empirical therapy before definitive culture results are validated [33]. By integrating structured culture follow-up patterns into predictive modeling, we facilitate earlier targeted therapy, consistent with current expert recommendations for managing high-risk patients [33].

This acceleration is technically grounded in the operational mechanics of automated systems such as VITEK^®^ 2. According to the manufacturer’s specifications, while species identification is typically achieved within 5–8 h, the final AST profile for an isolate can take up to 18 h due to variable bacterial growth kinetics. In a real-world simulation, our model utilizes the initial kinetic data available by hour 6 to predict the resistance status of delayed-reporting agents like carbapenems or colistin. This provides a critical 10–12 h lead time, allowing clinicians to initiate targeted therapy long before the final laboratory validation is completed.

Recent work has shown major improvements in ML-based antibiotic resistance prediction performance (Table 4), driven by diverse datasets, powerful ensemble methods (e.g., RF, XGBoost, LightGBM), deep learning techniques, and optimized preprocessing/validation strategies [30,31,34,35,36,37,38,39,40,41]. Our findings are consistent with these advances. Compared with previous studies focusing on limited antibiotic panels or ICU-restricted populations, our comprehensive model predicted resistance to 22 antibiotics using routine clinical and microbiological metadata. The RF model achieved an overall AUC of 0.96 and near-perfect predictive performance for carbapenems-ertapenem (100%), imipenem (93%), and meropenem (95%). Moreover, as summarized in Table 4, our model achieves one of the highest predictive performances reported to date (AUC: 0.96), comparable to studies using MALDI-TOF MS-based ML or national surveillance datasets for carbapenem-resistant *Klebsiella pneumoniae*. Importantly, unlike investigations restricted to single-drug outcomes or narrow antibiotic panels (e.g., carbapenems or ceftazidime-avibactam alone), our approach provides broad-spectrum resistance prediction across 22 antibiotics using only routinely collected clinical and microbiological metadata [13,14,15,30,31]. This combination of high performance, broad coverage, and practical feasibility distinguishes our work from prior ML studies and enhances its potential for real-world implementation across diverse healthcare settings [13,14,15,30,31,36,38,41,42].

Our findings echo the high predictive performance reported by Jian et al. [11], who achieved AUC values of 0.96–0.98 using Random Forest models. However, we diverge significantly in application scope. While prior work largely concentrated on high-priority phenotypes like carbapenem and colistin resistance, we maintained this high accuracy (AUC: 0.96) across a much broader spectrum of 22 antibiotics. This expansion is deliberate; it enables the model to guide decisions not just for MDR cases, but also for routine therapies involving narrower-spectrum agents such as cephalosporins.

Beyond standard metrics like Accuracy, the true value of this tool rests on its safety profile. Recognizing that a “false susceptible” error (Type II) carries far more risk than a false resistance prediction, we deliberately prioritized sensitivity over raw precision. By calibrating thresholds via Youden’s J statistic, we achieved high sensitivity for critical agents, 0.97 for Ertapenem and Cefazolin, and 0.94 for Meropenem (Table 3). Moreover, with NPVs exceeding 0.90 for carbapenems, the model offers the high confidence needed for antimicrobial stewardship, potentially empowering clinicians to de-escalate treatment safely.

Furthermore, the interpretability analysis revealed that resistance to one antibiotic strongly influenced the prediction of resistance to others, highlighting multi-drug resistance linkages likely driven by plasmid-mediated co-selection mechanisms. These interactions are clearly visualized in Figure 2, which displays the permutation feature importance landscape across antibiotic-specific models. Numerical feature ranking details are available in the Supplementary File “Feature Importance Table”. Beyond expected correlations within pharmacological classes, our model identified cross-class influences that reflect the complex biology of resistance evolution and may inform the redesign of AST panels. Antibiotics with strong mutual impact could be deprioritized to reduce unnecessary testing in ML-assisted workflows.

Our feature importance analysis revealed that, beyond inter-antibiotic correlations, clinical and demographic variables such as sample collection year, hospital department, and gender played distinct roles in predicting resistance. The emergence of ‘Year’ as a predictive feature is particularly significant; it likely reflects the dynamic evolution of antimicrobial resistance, capturing temporal trends such as the dissemination of specific high-risk clones or shifts in hospital antibiotic consumption policies over the study period. Similarly, the contribution of ‘Department’ highlights the heterogeneity of selection pressure across different hospital wards. For instance, isolates from intensive care units often exhibit distinct resistance profiles compared to those from outpatient clinics due to differences in patient acuity, length of stay, and invasive device usage. Interestingly, gender also contributed to the model’s decision-making for certain antibiotics. While biological mechanisms are not directly causative, this association may be driven by differential patterns of healthcare exposure and prior antibiotic prescriptions, such as the higher frequency of empirical treatment for urinary tract infections in females, which can differentially shape the host microbiota’s resistome over time.

Implementation of such ML-supported diagnostic tools could substantially reduce laboratory workload, shorten time to optimal therapy, and minimize unnecessary broad-spectrum antibiotic exposure. Ultimately, this supports reducing hospital stays and significantly lowering the economic burden associated with *Klebsiella pneumoniae* infections by minimizing the use of expensive broad-spectrum antibiotics and facilitating earlier modification of isolation precautions [26,27]. Given the rising global prevalence of resistant *Klebsiella pneumoniae* and the limited number of studies addressing broad-range resistance prediction, the comprehensive scope of our analysis provides an important contribution to both infectious diseases and ML-driven antimicrobial stewardship.

Beyond individual patient predictions, the proposed machine learning framework can offer a broader systemic benefit by operating continuously in the background as a dynamic surveillance tool. Unlike traditional antibiograms, this model can analyze real-time data flow to detect subtle, emerging shifts in resistance patterns that may escape human observation. Moreover, unlike genomic-based surveillance systems that require costly sequencing infrastructure, this approach utilizes routinely collected clinical data, offering a scalable and cost-efficient alternative, particularly suitable for resource-limited healthcare settings. By capturing fluctuating epidemiological trends without additional laboratory expenditure, the system provides a data-driven basis for adapting empirical therapy protocols promptly, which may mitigate the economic burden associated with therapeutic failures and prolonged hospitalizations. However, it is important to note that the full realization of these clinical and economic benefits requires further validation on larger, multi-center datasets. Future studies should focus on the prospective deployment of such models to quantify their actual impact on cost reduction and patient outcomes in diverse healthcare settings.

Despite these strengths, this study has limitations. Our model was developed using retrospective and single-center data, which may restrict external validity. Temporal split validation partially mitigates data leakage risk, but resistance patterns and laboratory practices vary regionally. Therefore, validation using multicenter, prospective cohorts is critical to confirm model robustness and ensure reliable deployment in diverse healthcare settings. Although SMOTE and imputation techniques were employed to address data balance and completeness, stringent cross-validation was applied to minimize synthetic bias. The high consistency between training and test results supports the validity of this approach despite the single-center design. Future work incorporating genomic and proteomic features could further improve predictive accuracy and provide mechanistic insights into co-resistance and virulence pathways.

## 5. Conclusions

This study demonstrates that explainable machine-learning models can provide rapid and accurate predictions of antimicrobial resistance in *Klebsiella pneumoniae* using routinely available clinical and microbiological data. The Random Forest classifier achieved high predictive performance, including near-perfect accuracy for carbapenem resistance, while inferring broad-spectrum resistance profiles from a limited subset of early AST results. This approach offers a complementary decision-support tool that may shorten time to optimal therapy, reduce unnecessary broad-spectrum antibiotic use, and lower healthcare costs. Further validation in multicenter, prospective cohorts is warranted to confirm generalizability and support integration into routine microbiology workflows.

## Figures and Tables

**Table 1:** Demographics of study participants (*n* = 607).

Characteristics		n	(%)
Gender	Male	331	54.5
	Female	276	45.5
Sample Year	2017	36	5.93
	2018	83	13.7
	2019	77	12.7
	2020	81	13.3
	2021	115	18.9
	2022	126	20.8
	2023	89	14.7
Sample Clinics	Adult (Cardiology and Cardiovascular Surgery (C&CVS))	353	58.2
	Child (C&CVS)	114	18.8
	Other ^1^	140	23.1
Sample Type	Wound	172	28.3
	Urine	147	24.2
	Tracheal Aspirate Culture	135	22.2
	Blood	71	11.7
	Phlegm	34	5.6
	Other ^2^	48	7.91

^1^ internal medicine, urology, gynecology, emergency operating room, anesthesia and reanimation polyclinic. ^2^ tissue biopsy, pleural fluid, peritoneal fluid.

**Table 2:** List of antibiotics used in the study and sample numbers according to their resistance status.

Group	Antibiotic Name	Resistance Status (Total *n* = 607)		
		Resistant n (%)	Intermediate Susceptibility n (%)	Susceptible n (%)
Aminoglycosides	Amikacin	247 (40.7%)	40 (6.6%)	320 (52.7%)
	Gentamicin	325 (53.5%)	6 (1.0%)	276 (45.5%)
Carbapenems	Ertapenem	330 (54.4%)	0 (0.0%)	277 (45.6%)
	Imipenem	325 (53.5%)	10 (1.6%)	272 (44.8%)
	Meropenem	269 (44.3%)	38 (6.3%)	300 (49.4%)
Cephalosporins	Cefazolin	450 (74.1%)	23 (3.8%)	134 (22.1%)
	Cefepime	424 (69.9%)	9 (1.5%)	174 (28.7%)
	Cefixime	426 (70.2%)	0 (0.0%)	181 (29.8%)
	Cefoxitin	358 (59.0%)	0 (0.0%)	249 (41.0%)
	Ceftazidime	444 (73.1%)	6 (1.0%)	157 (25.9%)
	Ceftriaxone	448 (73.8%)	0 (0.0%)	159 (26.2%)
	Cefuroxime	456 (75.1%)	51 (8.4%)	100 (16.5%)
	Cefuroxime axetil	460 (75.8%)	0 (0.0%)	147 (24.2%)
Fluoroquinolones	Ciprofloxacin	387 (63.8%)	16 (2.6%)	204 (33.6%)
Nitrofurans	Nitrofurantoin	416 (68.5%)	7 (1.2%)	184 (30.3%)
Other	Fosfomycin	400 (65.9%)	0 (0.0%)	207 (34.1%)
Penicillins and Beta Lactamase Inhibitors	Amoxicillin/clavulanic acid	456 (75.1%)	0 (0.0%)	151 (24.9%)
	Ampicillin	600 (98.8%)	0 (0.0%)	7 (1.2%)
	Piperacillin/tazobactam	393 (64.7%)	33 (5.4%)	181 (29.8%)
Polymyxins	Colistin	190 (31.3%)	0 (0.0%)	417 (68.7%)
Sulfonamides	Trimethoprim/sulfamethoxazole	368 (60.6%)	0 (0.0%)	239 (39.4%)
Tetracyclines	Tigecycline	321 (52.9%)	39 (6.4%)	247 (40.7%)

Note: Resistance rates were calculated based on the total number of isolates (*n* = 607). Susceptibility testing was performed and interpreted according to EUCAST (European Committee on Antimicrobial Susceptibility Testing) standards, which are the mandatory national standard in Türkiye.

**Table 3:** Performance metrics of the best performing RF model for each antibiotic resistance classification.

Algorithm	Antibiotic Name	Accuracy	Precision	Recall	F Score	AUC
RF Model	Amikacin	0.82	0.83	0.82	0.82	0.93
	Amoxicillin/clavulanic acid	0.93	0.87	0.77	1.00	0.98
	Ampicillin	0.98	0.87	0.86	0.88	0.98
	Cefazolin	0.97	0.97	0.97	0.97	0.99
	Cefixime	0.98	0.96	0.92	1.00	1.00
	Cefoxitin	0.93	0.92	0.98	0.86	0.99
	Ceftazidime	0.96	0.95	0.96	0.95	0.93
	Ceftriaxone	0.98	0.95	0.94	0.97	0.98
	Cefuroxime	0.96	0.96	0.96	0.96	0.96
	Cefuroxime axetil	1.00	1.00	1.00	1.00	1.00
	Cefepime	0.95	0.94	0.95	0.94	0.96
	Ciprofloxacin	0.89	0.87	0.89	0.88	0.93
	Colistin	0.72	0.79	0.73	0.85	0.78
	Ertapenem	0.98	0.98	0.97	1.00	1.00
	Fosfomycin	0.93	0.90	0.92	0.88	0.98
	Gentamicin	0.85	0.85	0.85	0.85	0.96
	Imipenem	0.93	0.93	0.93	0.93	0.93
	Meropenem	0.94	0.94	0.94	0.94	0.95
	Nitrofurantoin	0.93	0.92	0.93	0.92	0.97
	Piperacillin/tazobactam	0.89	0.89	0.89	0.89	0.98
	Tigecycline	0.87	0.86	0.87	0.87	0.94
	Trimethoprim/sulfamethoxazole	0.84	0.79	0.77	0.82	0.91
	MEDIAN	0.92	0.87	0.86	0.88	0.96

**Table 4:** Comparative table of studies on ML-based antimicrobial resistance prediction.

No	Study (Year)	Country	Data Type	Number of Samples	Data Collecting Year	ML Algorithms Used	Performance Metrics (AUC)	Best Model	Study Aim
1	Sullivan et al. (2018) [39]	USA	Electronic Medical Records, pneumonia and bacteremia cases.	613	2012–2016	MLR	AUC: 0.73	MLR	Carbapenem resistance prediction in patients with *Klebsiella pneumoniae*
2	Feretzakis et al. (2020) [30]	Greece	Demographic info, culture results, drug susceptibility, Gram-stain.	345	2017–2018	LIBLINEAR; LIBSVM; SMO; kNN-5; J48; RF; RIPPER; MLP	AUC: 0.568–0.726	MLP	Empirical antibiotic therapy decisions in intensive care units
3	Lewin-Epstein et al. (2021) [41]	Israel	Electronic medical records.	2347	2013–2015	LASSO LR; NN; GB; Ensemble	AUC: 0.80–0.88	Ensemble	Predicting antibiotic resistance
4	Feretzakis et al. (2021) [31]	Greece	Clinical data.	2131	2019	JRip; RF; MLP; Classification Regression; REPTree	AUC: 0.865–0.933	Classification Regression	Predict antimicrobial resistance
5	Pascual-Sánchez et al. (2021) [34]	Spain	Electronic health records.	3476	2004–2020	LR; DT; RF; XGBoost; MLP	AUC: 0.76	LR, XGBoost	Predicting multidrug resistance
6	Liang et al. (2022) [35]	China	Clinical, demographic, medical history data.	2920	2017–2021	RF; XGBoost; DT; LR	AUC: 0.78–0.91	Strong models	Prediction of carbapenem-resistant Gram-negative bacterial carriage
7	Corbin et al. (2022) [36]	USA	Electronic health records, drug susceptibility.	6920	2009–2019	LASSO; Ridge LR; RF; GB	AUC: 0.64–0.74	Moderate accuracy	ML-driven antibiotic selection
8	Tzelves et al. (2022) [37]	Greece	Urine samples, demographic, culture, susceptibility, Gram-stain.	239	2019	MLR	AUC: 0.77–0.87	Good discriminator	Predict antimicrobial resistance in stone disease patients
9	Wang et al. (2022) [40]	China	MALDI-TOF MS spectral data.	171	2020–2021	RF; Nonlinear SVM; SVM-K	AUC: 0.936	SVM-K	Rapid Detection of Carbapenem-Resistant *Klebsiella pneumoniae*
10	Zeng et al. (2023) [42]	China	Clinical data of isolates.	49,774	2018–2021	LR; ANN	AUC: 0.837	ANN	Predicting Carbapenem-resistant *Klebsiella pneumoniae*
11	Mintz et al. (2023) [38]	Israel	Electronic health records.	357	2016–2019	Ensemble (LASSO LR; RF; GB; NN); Stacked learner (LR)	AUC: 0.737–0.837	Ensemble	Prediction of ciprofloxacin resistance
12	Lin et al. (2024) [15]	China	MALDI-TOF MS and antimicrobial susceptibility testing data.	675	2022–2023	LR; LDA; RF; GB; AdaBoost; XGBoost; LightGBM	AUC: 0.95	LightGBM	Detection of Ceftazidime-avibactam resistance in *Klebsiella pneumoniae*
13	Jian et al. (2024) [11]	China	Multidrug resistance *Klebsiella pneumoniae* data.	4307	2022	LR; LDA; RFC; GBC; ABC; XGBoost; LightGBM; SVM	AUC: 0.96–0.98	RFC	Prediction of carbapenem and colistin resistant *Klebsiella pneumoniae*
14	Pan et al. (2025) [14]	China	Patient data with multidrug-resistant *Klebsiella pneumoniae* infection.	1385	2019–2024	LR; DT; RF; XGBoost; SVM; KNN; LightGBM	AUC: 0.906	LR	Predict multidrug-resistant *Klebsiella pneumoniae* -related septic shock
15	Alparslan et al. (2025) [13]	Türkiye	Demographic, clinical, laboratory data.	289	2017–2023	XGBoost; LR; RF; SVM; LightGBM; MLP; DT	AUC: 0.91	XGBoost	Predict carbapenem-resistant *Klebsiella pneumoniae* infection in ICU patients
16	Our Study (2025)	Türkiye	Demographic, clinical, antibiotic susceptibility testing data.	607	2017–2024	DT; SVC; KNN; RF	AUC: 0.96	RF	Rapid prediction of resistance to 22 antibiotics in *Klebsiella pneumoniae* isolates

## Data Availability

The patient-based antibiogram data are not publicly available due to ethical and privacy restrictions. De-identified data underlying the findings of this study will be shared as a Supplementary File (Study Data) in accordance with institutional and national ethical regulations.

## Supplementary Materials

The following supporting information can be downloaded at: https://www.mdpi.com/article/10.3390/diagnostics16040555/s1, Supplementary File: comprehensive dataset, machine learning algorithm performance results, and feature importance rankings (excel file containing three sheets: “Study Data”, “Algorithm Results”, and “Feature Importance Table”).

## Abbreviations

The following abbreviations are used in this manuscript:

ABC	AdaBoost Classifier
AdaBoost	Adaptive Boosting
ANN	Artificial Neural Network
DT	Decision Tree
GB	Gradient Boosting
J48	Decision Tree Classifier
JRip	Rule learner for classification
KNN	k-Nearest Neighbors
LASSO	Least Absolute Shrinkage and Selection Operator
LDA	Linear Discriminant Analysis
LIBLINEAR	Library for Large Linear Classification
LIBSVM	Library for Support Vector Machines
LightGBM	Light Gradient Boosting Machine
LR	Logistic Regression
MLP	Multilayer Perceptron
MLR	Multiple Logistic Regression
NN	Neural Network
RF	Random Forest
RFC	Random Forest Classifier
RIPPER	Repeated Incremental Pruning to Produce Error Reduction
SMO	Sequential Minimal Optimization
SVC	Support Vector Classifier
SVM	Support Vector Machine
SVM-K	Support Vector Machine with Dimension Reduction
XGBoost	eXtreme Gradient Boosting
